# Indole-Derived Compounds as Redox-Modulators: Antioxidant Mechanisms in Neuronal Protection

**DOI:** 10.3390/molecules31132323

**Published:** 2026-07-02

**Authors:** Alka Ashok Singh, Ananta Prasad Arukha, Minseok Song

**Affiliations:** 1 Department of Life Sciences, Yeungnam University, Gyeongsan 38541, Republic of Korea; 2 Department of Infectious Disease and Immunology, University of Florida, Gainesville, FL 32608, USA

**Keywords:** indole derivatives, oxidative stress, neuroprotection, redox modulation, neurodegenerative diseases, Nrf2/ARE signaling, melatonin, indole-3-carbinol, indole-3-propionic acid, neuroinflammation

## Abstract

Neurodegenerative diseases, such as Alzheimer’s, Parkinson’s, and Amyotrophic lateral sclerosis, are distinguished by progressive neuronal dysfunction caused primarily by oxidative stress, mitochondrial impairment, neuroinflammation, and redox imbalance. Growing evidence suggests that indole-derived compounds have significant neuroprotective potential due to their antioxidant, anti-inflammatory, and redox-modulating properties. This review summarizes the structural and biological significance of indole scaffolds, focusing on the mechanisms by which natural, endogenous, microbiota-derived, and synthetic indole compounds protect neuronal networks. Indole-3-carbinol, 3,3′-diindolylmethane, indole-3-propionic acid, and melatonin are major indole derivatives that control important neuroprotective pathways like Nrf2/ARE signaling, mitochondrial bioenergetics, neurotrophic factor expression, apoptotic regulation, and suppression of proinflammatory mediators. These compounds also maintain synaptic plasticity, reduce reactive oxygen species production, and improve neuronal survival in neurodegenerative disease models. Additionally, updated information from translational and clinical research indicates that indole-based compounds may have promising therapeutic applications; however, obstacles like low bioavailability, metabolic instability, and blood–brain barrier penetration continue to be major obstacles to clinical application. Development in nanoparticle delivery systems, microbiome-targeted interventions, and rational structural optimization may improve therapeutic efficacy and translational potential. Overall, indole-derived compounds are a versatile class of redox modulators with potential applications in the prevention and treatment of neurodegenerative diseases via integrated antioxidant and neuroprotective mechanisms.

## 1. Introduction

Neurodegenerative diseases (NDs) are characterized by the progressive degeneration of neurons, synapses, glial cells, and their associated networks. These diseases can be classified based on: (1) their predominant clinical manifestations, such as dementia, Parkinsonism, or motor neuron disease; (2) the anatomical distribution of neurodegeneration, such as frontotemporal degeneration, extrapyramidal disorders, or spinocerebellar degeneration; or (3) the principal molecular abnormalities [1]. The prevalence of neurodegenerative disorders is increasing among older adults, with aging recognized as the primary risk factor. Disorders such as amyotrophic lateral sclerosis (ALS), Huntington’s disease (HD), Parkinson’s disease (PD), Alzheimer’s disease (AD), and spinocerebellar ataxia (SCA) are characterized by progressive neuronal loss, abnormal protein aggregation, and impaired motor or cognitive function [2]. There is growing evidence that oxidative stress and redox imbalance play a crucial role in the development and progression of these conditions [3]. Excessive accumulation of reactive oxygen species (ROS) and other free radicals, frequently resulting from mitochondrial dysfunction, impaired antioxidant defenses, or environmental stressors, causes oxidative damage to proteins, lipids, and nucleic acids [4]. Such damage disrupts cellular homeostasis, promotes neuroinflammation, and accelerates neuronal death, thereby creating a vicious cycle that drives disease progression [5].

Inflammation is a fundamental defensive response to harmful stimuli; however, it becomes dysregulated and overactivated in most human diseases. At moderate levels, ROS, generated through incomplete reduction in molecular oxygen, act as critical signaling molecules in modulating various physiological functions, including inflammatory responses. However, excessive ROS levels exert toxic effects by oxidizing biological macromolecules like proteins, nucleic acids, and lipids, thereby aggravating inflammatory responses and contributing to diverse inflammatory diseases [6]. Living cells produce ROS as a normal byproduct of cellular metabolism. Under stress, excessive ROS production prompts cells to activate adaptive response mechanisms that utilize ROS as signaling molecules. ROS molecules can induce oxidative stress through a feedback mechanism involving multiple biological processes, including necrosis, autophagy, and apoptosis [7]. Consequently, recent developments have focused on modulating redox signaling pathways to restore redox homeostasis, enhance cellular resilience, and lower oxidative stress. This targeted approach preserves ROS’s regulatory and protective roles in neuronal systems, thereby offering greater therapeutic potential.

**Figure 1:**
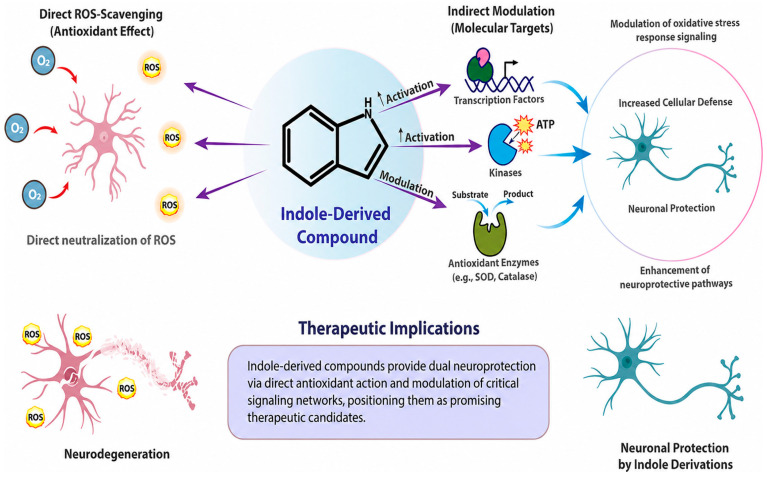
Schematic overview illustrating the role of oxidative stress and redox imbalance in neurodegenerative diseases, and the neuroprotective properties of indole-derived compounds.

Indole-derived compounds exhibit tremendous potential in neuroprotection because of their unique chemical scaffolds, which confer potent antioxidant activity and the capacity to modulate redox-sensitive signaling pathways. Recently, indole and its aromatic heterocyclic derivatives have gained significant attention in studies on NDs [8,9]. Several synthetic indole-phenolic compounds have been evaluated as multifunctional neuroprotectors based on the established neuroprotective effects of indole-based compounds and their potential for multitargeted therapy. Every compound exhibited metal-chelating qualities, especially toward copper ions, with quantitative analysis demonstrating approximately 40% chelating activity for each compound. In cellular models, these hybrid compounds countered ROS produced by the Aβ (25–35) peptide and its oxidative byproduct, hydrogen peroxide, as evidenced by quantitative analysis that showed an average 25% increase in cell viability and restoration of ROS levels to basal states [10]. Indole derivatives are abundant in natural compounds and play key roles in both biological and medicinal systems. Indole is a highly conserved molecule with broad-spectrum antioxidant activity and scavenges free radicals [11]. Additionally, indole and its derivatives exhibit diverse medicinal benefits, including immune system and circadian rhythm regulation [12]. Due to its high resonance stability and extremely low activation energy barrier toward free radical reactions, the indole ring in the melatonin molecule acts as the primary reactive moiety against oxidants. Indole-3-propionamide derivatives exhibit greater antioxidant activity than melatonin, while 2-phenylindole derivatives considerably reduce lipid peroxidation [13]. The indole skeleton has demonstrated protective effects in various in vitro and in vivo oxidative stress models. Indoles have also been shown to enhance GSH production, increase GPx activity, and inhibit lipid peroxidation through direct scavenging of ROS [13]. Beyond their ROS-scavenging properties, these compounds interact with molecular targets, such as transcription factors, kinases, and oxidative stress-related enzymes, thereby enhancing endogenous neuroprotective pathways. Indole derivatives combat neurodegeneration through both direct antioxidative activity and modulation of key signaling networks, highlighting their potential as promising therapeutic agents (Figure 1).

Despite mounting evidence that indole-derived compounds have neuroprotective properties, there are still numerous important information gaps. First, there has been no comprehensive assessment of the molecular processes behind the neuroprotective activities of particular indole derivatives across a wide range of neurodegenerative diseases. Second, little attention has been devoted to failed translational and clinical outcomes, impeding a comprehensive understanding of the reasons limiting therapeutic efficacy. Furthermore, a thorough comparison of natural, endogenous, microbiota-derived, and synthetic indole derivatives within a single redox-modulation framework has seldom ever been included in prior reviews. Additionally, the clinical translation of many promising indole-based drugs is still hampered by low bioavailability, fast metabolism, and limited blood–brain barrier penetration. It is anticipated that future clinical research will concentrate on enhancing pharmacokinetic characteristics, maximizing brain delivery, and finding biomarkers that can precisely assess neuroprotective activity. To improve the clinical translation of indole-based neuroprotective techniques and guide future therapeutic research, it is imperative to close these gaps.

## 2. Indole Framework and Biological Relevance

The indole moiety, composed of fused pyrrole and benzene rings, is a key structural component in organic chemistry due to its widespread presence in both natural and synthetic compounds. Indole plays a crucial role in bioactive molecules, including alkaloids, hormones, and pharmaceuticals, making it significant in medicinal chemistry, material science, and other fields. Its unique reactivity and structural versatility enable the synthesis of complex molecules, facilitating innovative drug design and development [14,15]. Recently, the indole core has emerged as a versatile scaffold in drug research. Although initially identified for its anticancer activity in vinca alkaloids [16], indole has since demonstrated therapeutic potential in diverse diseases, including diabetes, HIV, Alzheimer’s, and hyperlipidemia. Due to their biochemical diversity across plants, bacteria, animals, marine organisms, and humans, as well as their presence in multiple FDA-approved medications, indole derivatives have become valuable compounds in pharmaceutical research [17].

### 2.1. Structural Features and Chemical Diversity

Indole is a privileged heterocyclic scaffold consisting of a benzene ring fused to a pyrrole moiety [18]. Its electron-rich, planar structure readily undergoes substitution at multiple locations (N1, C2, C3, and throughout the aromatic ring), yielding extensive chemical diversity [19]. Such versatility accounts for the broad spectrum of biological functions exhibited by indole derivatives [19]. Importantly, modifications at key positions alter electron distribution and radical-scavenging properties, enabling fine-tuning of antioxidant activity and interactions with redox-sensitive signaling pathways involved in neuroprotection. Several curcumin-coumarin hybrids have been developed based on the known neuroprotective and antioxidant potentials of simple coumarins. The inhibition of other AD-correlated protein kinases, like CK1 and LRRK2, has recently emerged as a promising therapeutic strategy, with indole identified as a valuable scaffold for inhibiting both kinases. To generate useful BBB-permeable pharmacological tools, a small library of indole-based derivatives was created. To prepare nanoparticles for novel drug-delivery and targeting systems, chitosan (CS), a naturally occurring, nontoxic, biocompatible, and biodegradable polysaccharide, was selected to create CS-based bioconjugates [20].

### 2.2. Natural and Endogenous Sources:

The indole structure, including indole and its substituted derivatives, has attracted significant scientific interest because of its diverse biological activities. Indole-containing heterocycles are widely recognized for their importance in the fields of medicinal chemistry, drug design and discovery, agrochemicals, photochemistry, dyes, and other applications [21].

Upon digestion, these compounds undergo enzymatic or acidic transformation into bioactive metabolites with antioxidant and anti-inflammatory effects.

Endogenous Metabolites: Endogenous metabolite concentrations can be altered through various mechanisms. Inborn errors of metabolism, a large group of rare genetic human diseases, are characterized by significant derangements of single endogenous small molecule metabolites, many of which are associated with prominent neuropsychiatric symptoms. Whether such metabolites exert neuroactive effects to directly cause neural dysfunction has been widely speculated, but conclusive evidence remains limited [22]. Tryptophan (Trp) metabolism produces several neurobiologically relevant indole derivatives, including serotonin, melatonin, and kynurenine pathway metabolites [23]. Beyond their role in regulating circadian and neurotransmitter functions, these compounds also help maintain redox homeostasis in the central nervous system (CNS) [24]. Within the CNS, L-tryptophan metabolism is a highly controlled physiological process that generates multiple neuroactive substances, including the neurohormone melatonin, neuroactive melatonin-derived kynuramine metabolites, trace quantities of tryptamine, aminergic neurotransmitter serotonin (5-hydroxytryptamine, 5-HT), and products of the kynurenine pathway of tryptophan metabolism, such as 3-hydroxykynurenine, 3-hydroxyanthranilic acid, quinolinic acid, and kynurenic acid [25].

Microbiota-Derived Indoles: The gut–brain axis represents a crucial source of redox-active indoles because the gut microbiome generates a varied range of indole derivatives that can affect neuronal redox signaling and systemic inflammation [26]. In recent years, gut–brain axis signaling has emerged as a key regulator of mood, behavior, cognition, and cellular viability under both physiological and pathological settings. Therefore, the intestinal microbiome has become a promising therapeutic target for psychiatric and neurological conditions. Indole-3-propionic acid (IPA), a microbiota-derived metabolite of Trp, has been shown to influence brain function and modulate multiple molecular processes. In this review, we outline the main mechanisms through which IPA may influence neuronal survival and activity and provide an update on evidence demonstrating its neuroprotective effects across a range of experimental settings [27]. Research indicates that Trp metabolites considerably attenuate age-related neurodegeneration through the GPR30/AMPK/SIRT1 pathway. This research offers a novel therapeutic strategy and target to mitigate neurodegeneration [28].

### 2.3. Synthetic Analogs

Because it is electron-rich and tolerant of diverse substitutions, the indole scaffold provides a flexible platform for designing synthetic analogs. To improve pharmacokinetic and pharmacodynamic properties, structural modifications at locations like C2, C3, and N1 have been extensively explored. In CNS drug development, such modifications fine-tune lipophilicity, metabolic stability, and blood–brain barrier permeability [29]. In medicinal chemistry, the indole scaffold is a highly valued structural motif recognized for its superior chemical adaptability, biological ubiquity, and clinical significance. This review provides a comprehensive overview of current studies on the indole nucleus, highlighting its physicochemical properties, reactivity patterns, and ability to interact with multiple biological targets [29]. A central feature of indole chemistry is its electron-rich pyrrole ring. Medicinal chemistry can investigate wide-ranging structure-activity relationships because of the indole scaffold’s chemical versatility at various positions while preserving the core. The significance of the ring has led to the development of many synthetic pathways to indoles. Traditional named reactions, some dating back to the 19th and early 20th centuries, produce indole from different precursors [29]. The classic example is the Fischer indole synthesis, in which acid catalysis converts aryl hydrazones to indoles. Tryptamine derivatives and indole-3-acetic acid are industrially synthesized through several Fischer-based processes. Other classical routes for indole production include the Bischler-Möhlau (from α-halo-ketones and anilines), Baeyer-Emmerling (from orthonitrocinnamic acids), Madelung (intramolecular cyclization of N-phenylamides), Reissert (cyclization of α-aryl-β-aminoketones), Bartoli (addition of vinyl Grignard to nitroanilines), Larock (Pd-catalyzed annulation of anilines with alkynes), and Leimgruber-Batcho syntheses. Each method provides distinct entry points depending on the available starting materials [30,31]. Strategic substitution patterns can augment metabolic stability, hydrophobic-hydrophilic balance, and blood–brain barrier penetration, thereby increasing therapeutic potential in neurodegenerative disorders [32]. The indole framework is incorporated into several CNS-active medications, leveraging its capacity to interact with melatonin, serotonin, and other neural pathways. As noted previously, serotonergic systems are the primary focus. The indole core was present in the early antidepressant indalpine, a selective 5-HT reuptake inhibitor first used in France in the 1980s [33]. Indoles have been investigated as neuroprotective agents in neurodegenerative illnesses. Among tryptophan metabolites, indole-3-propionic acid (IPA), a potent hydroxyl radical scavenger, has demonstrated protective effects in models of AD and HD, whereas kynurenic acid, despite not being an indole, also exhibits neuroprotective properties [34,35,36].

### 2.4. Pharmacokinetics and Blood–Brain Barrier Permeability

**Figure 2:**
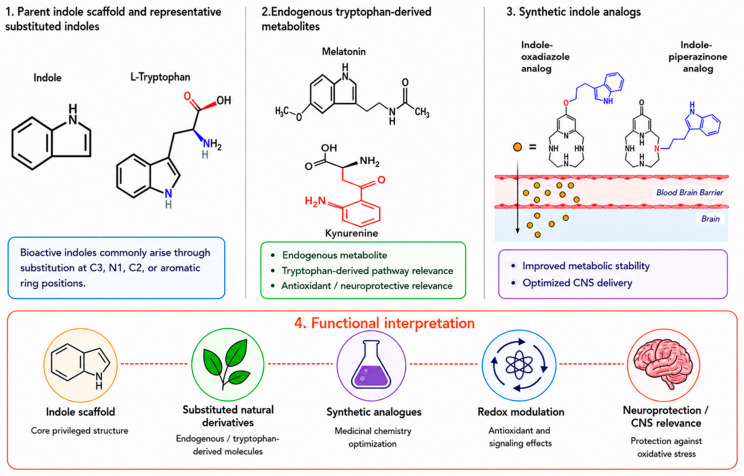
Indole-derived compounds: structural features, biological sources, and pharmacological relevance.

One of the biggest challenges in the development of drugs is predicting how well they will penetrate the human BBB. Although a number of in vitro systems that mimic the blood–brain barrier have been described, it is still unclear how best to extrapolate these findings to human unbound brain concentration profiles. To estimate the permeability and efflux of drugs across the blood–brain barrier, physiologically based pharmacokinetic (PBPK) modeling of drug disposition in the central nervous system (CNS) currently involves fitting preclinical in vivo data to compartmental models [37]. For indole derivatives to exert neuroprotective actions, effective CNS delivery is essential [38]. For indole derivatives to have neuroprotective effects, they must be delivered to the central nervous system effectively. However, brain absorption is determined by polarity, ionization state, metabolic stability, and blood–brain barrier permeability. Synthetic indole analogs are frequently optimized to balance solubility, metabolic stability, and CNS delivery. Figure 2 summarizes the structural and functional diversity of indole-derived chemicals. The figure distinguishes the primary indole scaffold from substituted indoles, endogenous tryptophan-derived metabolites/catabolites, and synthetic indole analogs. Importantly, bioactive indole derivatives are not depicted as metabolites convergent to a central indole core; rather, their biological activity is controlled by substitution at C3, N1, C2, and aromatic-ring locations, which can change redox activity, metabolic stability, and CNS transport. However, the degree of brain uptake is determined by variables like polarity, ionization level, and metabolic stability [39]. While synthetic analogs frequently need to be strategically modified to balance solubility and stability with BBB permeability, endogenous indoles, such as melatonin, effectively cross the BBB [40]. Important pharmacokinetic properties of indole-derived compounds include gastrointestinal stability, oral bioavailability, metabolic stability, plasma protein binding, tissue distribution, BBB permeability, and phase I/II biotransformation [29,41]. Natural indole compounds exhibit diverse pharmacokinetic profiles [42]. For example, melatonin readily crosses the BBB but undergoes extensive hepatic metabolism, whereas indole-3-carbinol (I3C) is unstable under acidic conditions and is rapidly converted into bioactive condensation products such as 3,3′-diindolylmethane (DIM) [43]. In contrast, indole-3-propionic acid (IPA) demonstrates relatively high metabolic stability and systemic availability [44]. Synthetic indole derivatives are frequently optimized to enhance lipophilicity, metabolic stability, BBB penetration, and central nervous system exposure, thereby improving their therapeutic potential for neurological disorders [45,46]. Additionally, antioxidant activity may be increased or decreased by phase I (oxidation, hydroxylation) and phase II (glucuronidation, sulfation) metabolic transformations, highlighting the significance of pharmacokinetic optimization in drug design [47]. The major biological sources, structural diversity, and pharmacological relevance of indole-derived compounds are summarized in Figure 2.

Figure 2 illustrates the relationship between structural diversity and pharmacological behavior of indole derivatives. Endogenous molecules such as melatonin possess favorable BBB permeability and direct antioxidant activity, whereas microbiota-derived metabolites such as IPA contribute to neuroprotection through modulation of gut–brain signaling pathways. Synthetic analogs expand this chemical space by improving metabolic stability and optimizing CNS delivery. Together, these structural classes demonstrate how source and molecular architecture influence neuroprotective efficacy.

## 3. Redox Dysregulation in Neuronal Injury

**Figure 3:**
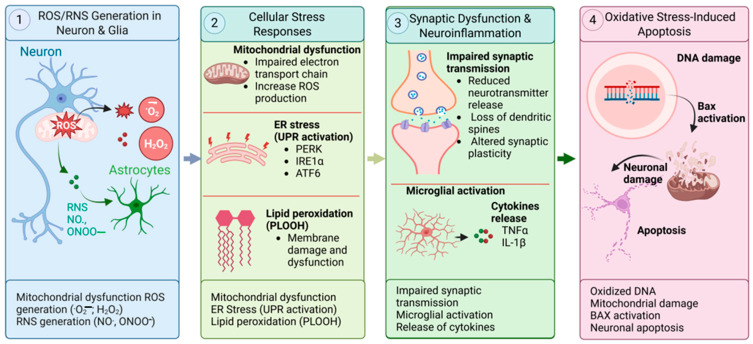
Molecular pathways underlying redox dysregulation and neuronal damage in neurodegenerative diseases. Reactive oxygen species (ROS) and reactive nitrogen species (RNS) are generated in neurons and glial cells due to mitochondrial dysfunction, oxidative stress, and glial activation. Excessive ROS/RNS production triggers cellular stress responses, including ER stress and lipid peroxidation, leading to synaptic dysfunction and neuroinflammation. Persistent oxidative damage promotes DNA damage, mitochondrial impairment, and neuronal apoptosis, ultimately contributing to neurodegeneration.

Cellular oxidation/reduction (redox) states preserve homeostasis and regulate various aspects of cellular activity [48]. Redox imbalance has been implicated in the onset and progression of several NDs [49]. The following subsections assess key mechanisms involved in redox imbalance-mediated neuronal injury, including the production of reactive oxygen and nitrogen species, mitochondrial and endoplasmic reticulum dysfunction, lipid peroxidation, altered synaptic plasticity and neuroinflammation, and the activation of apoptotic pathways (Figure 3).

### 3.1. Reactive Oxygen and Nitrogen Species (ROS/RNS) Generation in Neurons and Glia

Neurons are particularly susceptible to redox imbalance because of their high metabolic activity and substantial oxygen and energy demands [50]. In neurons, the primary sources of ROS include mitochondrial oxidative phosphorylation, NADPH oxidases, and enzymatic processes like monoamine oxidase activity [51]. ROS and reactive nitrogen species (RNS) are produced by glial cells, particularly microglia and astrocytes, as part of innate immune responses; however, excessive activation leads to chronic oxidative stress [52]. Overproduction of superoxide, hydroxyl radicals, nitric oxide, and peroxynitrite exceeds antioxidant defense capacity, disrupting cellular homeostasis and causing neuronal injury [53].

### 3.2. Mitochondrial Dysfunction, Endoplasmic Reticulum Stress, and Lipid Peroxidation

Mitochondria play a central role in redox regulation and are susceptible to oxidative stress-induced dysfunction [54]. They are essential hubs of energy production and redox regulation. Impairment of the mitochondrial electron transport chain results in electron leakage and ROS overproduction, causing mitochondrial dysfunction [55]. The mitochondrial genome is particularly prone to oxidative damage [56], and increased cellular ROS can further disrupt mitochondrial processes. Release of mitochondrial proteins, such as cytochrome c, into the cytoplasm may activate neuronal apoptotic signaling pathways [57]. There is strong evidence to indicate that neurodegeneration in both SOD1 rodents and mutant SOD1 overexpressed in cell culture is influenced by molecular components of mitochondrial apoptosis [58]. Another crucial site for ROS generation is the ER, which is redox-regulated. It is essential for protein and lipid synthesis as well as for the folding of proteins. The unfolded protein response (UPR), a unique signaling pathway intended to alleviate stress, is triggered by protein misfolding within the ER [59]. ER stress impairs protein folding, activating the UPR and ROS production, all of which contribute to redox dysregulation [60]. Lipid peroxidation products propagate oxidative damage by modifying proteins and nucleic acids, thereby exacerbating neuronal dysfunction. According to recent research, ROS can initiate lipid peroxidation (LPO) by attacking lipids with C-C double bonds, especially polyunsaturated fatty acids (PUFAs). This process generates toxic lipid aldehyde species, such as 4-hydroxy-2-nonenal (HNE), malondialdehyde, and acrolein. These reactive aldehyde species induce posttranscriptional modifications of proteins and DNA, ultimately leading to cytotoxicity, genotoxicity, impaired gene expression, and cellular death [61]. The CNS is a major target of LPO because neuronal tissue is especially vulnerable to free radical-mediated chain reactions that produce LPO products [62,63]. In addition to high oxygen consumption, the CNS contains elevated levels of PUFAs and redox transition metal ions, increasing its susceptibility to LPO [64]. Under conditions of excessive LPO, dopamine undergoes oxidative conversion to o-quinone, triggering a chain reaction. Subsequent intramolecular cyclization and molecular interaction with specific targets induce cytotoxic reactions and impaired cell function [65]. 4-hydroxynonenal (HNE), a hazardous byproduct of lipid peroxidation produced during oxidative stress, can modify proteins and disrupt cellular processes. Elevated HNE levels cause neuronal degeneration and functional decline in PD by contributing to dopamine depletion, toxic oligomer formation, and increased oxidative damage [61].

### 3.3. Oxidative Stress in Synaptic Plasticity and Neuroinflammation

Redox signaling plays a dual role in synaptic function. Physiological levels of ROS and RNS regulate synaptic plasticity by balancing long-term potentiation and long-term depression (LTD) [66,67]. Chronic oxidative stress, however, disrupts this balance, impairing synaptic signaling, decreasing dendritic spine density, and ultimately compromising learning and memory functions [68]. Oxidative stress also triggers microglial activation, triggering a neuroinflammatory cascade characterized by the release of proinflammatory cytokines such as TNF-α, IL-1β, and IL-6. This further exacerbates neuronal damage and creates a vicious cycle in which inflammation promotes additional ROS generation [69]. A basic mechanism for enhancing neural circuit function involves the restoration of key synaptic proteins (PSD-95, MAP2, and SYP). Increased Brain-derived neurotrophic factor (BDNF)-TrkB signaling, a pathway essential for neuroplasticity and antidepressant response, has also been reported [70].

### 3.4. Oxidative Stress-Induced Apoptosis

Evidence supporting the contribution of intracellular Ca^2+^ mediated altered homeostasis in the cascade of events leading to neuronal death should be considered in pathological settings where oxidative stress appears to play a significant role in producing neuronal injury. Under such conditions, intracellular Ca^2+^ overload happens during excitotoxicity, a mechanism of neuronal death caused by the overactivation of glutamate receptors [71,72]. When antioxidant defenses are overwhelmed, oxidative stress activates several proapoptotic pathways [73]. In addition to protein processing, the ER is involved in lipid synthesis, intracellular molecular transport, and the regulation of Ca^2+^ homeostasis. Before exiting the ER, proteins undergo folding and posttranslational modifications. Protein folding and revision require molecular chaperones and a favorable ER environment. Under stressful situations, alterations in ER luminal homeostasis or chaperone capacity activate signaling cascades that attempt proper protein folding through the UPR, which may also induce autophagy to preserve cell integrity. However, when the UPR is impaired or insufficient, cell death ensues [74]. Additionally, Endoplasmic reticulum (ER) stress has been linked to the pathophysiology of numerous diseases, including heart disease, cancer, and neurodegenerative diseases like Alzheimer’s and Huntington’s. Prolonged or excessive ER stress causes the initiation of signaling pathways, which leads to cell death [75]. Oxidative stress-driven apoptosis partly causes the progressive loss of neurons seen in NDs, highlighting the pathogenic role of redox imbalance.

Overall, these mechanisms illustrate how redox dysregulation contributes to multiple aspects of neuronal injury, including lipid peroxidation, synaptic impairment, neuroinflammation, apoptosis, mitochondrial dysfunction, and ER stress. Significantly, even though physiological ROS/RNS signaling is necessary for synaptic plasticity and neuronal communication, persistent overproduction of ROS/RNS triggers a pathological cascade that promotes neurodegeneration [66]. This study emphasizes the therapeutic significance of restoring redox balance. Because of their inherent antioxidant qualities and capacity to modulate redox-sensitive signaling pathways, indole-derived compounds show promise in preventing oxidative stress-induced neuronal damage.

## 4. Indole-Derived Compounds as Redox Modulators

**Figure 4:**
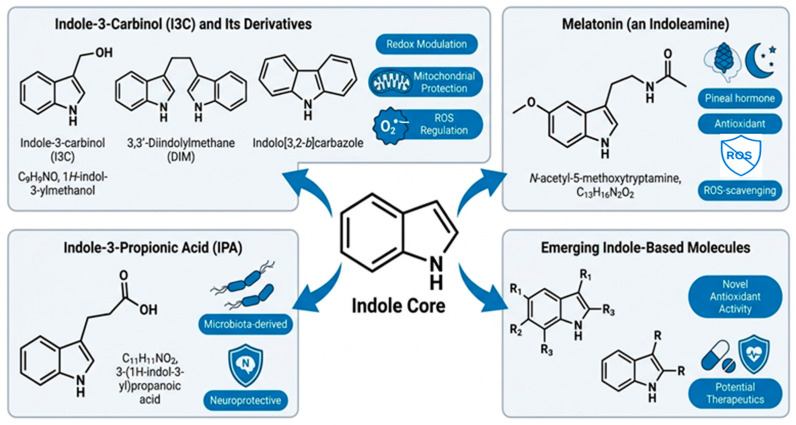
Overview of the neuroprotective and antioxidant roles of major indole-derived compounds centered around the indole core structure. Indole-3-carbinol (I3C) and its derivatives, including 3,3′-diindolylmethane (DIM) and indolo[3,2-b]carbazole, are associated with redox modulation, mitochondrial protection, and regulation of reactive oxygen species (ROS). Melatonin, an indoleamine produced by the pineal gland, exhibits antioxidant and ROS-scavenging activity. Indole-3-propionic acid (IPA), a microbiota-derived indole metabolite, contributes to neuroprotective defense against oxidative stress. Emerging indole-based molecules also demonstrate potential antioxidant activity and therapeutic relevance in neuroprotection.

In medicinal chemistry, the indole scaffold is a highly valued structural motif recognized for its remarkable chemical adaptability, biological ubiquity, and clinical significance [29]. Given the importance of indoles, several reviews over the past decade have investigated indole-containing compounds and their biological activities [46,76,77,78]. Notably, several dietary indole derivatives have demonstrated significant potential in regulating redox signaling pathways and oxidative stress. Representative indole-derived neuroprotective compounds and their mechanisms are illustrated in Figure 4. A comprehensive summary of the specific neuroprotective roles, source pathways, and targeted disease models for these major indole-derived compounds is provided in Table 1.

### 4.1. Indole-3-Carbinol (I3C) and Its Derivatives

Indole-3-carbinol (I3C), a common phytochemical found in cruciferous vegetables, and its condensation product, 3,3′-diindolylmethane (DIM), exhibit multiple cellular and molecular biological activities that contribute to their well-established chemopreventive potential. Initially, these substances were categorized as blocking agents because of their ability to enhance the activity of drug-metabolizing enzymes [86]. Following ingestion, I3C undergoes acid-catalyzed oligomerization in the stomach, producing bioactive derivatives like DIM. Through alteration of redox-sensitive transcription factors, I3C and DIM exert potent anti-inflammatory and antioxidant effects [87]. Specifically, expression of phase II antioxidant enzymes (such as glutathione S-transferases and heme oxygenase-1) is upregulated when the Nrf2/ARE pathway is activated [88], whereas inhibition of NF-κB signaling suppresses proinflammatory mediators, including TNF-α, IL-6, and COX-2 [89]. I3C derivatives demonstrate therapeutic promise in neurodegenerative contexts by lowering oxidative stress-induced apoptosis, augmenting mitochondrial function, and attenuating neuroinflammatory responses in neuronal cell culture and animal models [43]. According to a study, I3C significantly mitigates oxidative stress by increasing antioxidant enzymes like HO-1 and NRF2, reducing inflammatory markers including NF-kB, TNF-α, and IL-6, and increasing IL-10 levels. In addition, I3C helps restore cholinergic pathway function, underscoring its potential as a therapeutic strategy for improving cognitive deficits [90].

### 4.2. Indole-3-Propionic Acid (IPA)

Indole-3-propionic acid (IPA), a gut microbiota-derived metabolite of tryptophan, is a unique indole derivative with potent antioxidant properties [91]. It is mainly produced by bacteria, including species present in human intestinal microbiota and soil environments. The Clostridiaceae and Peptostreptococcaceae families are the primary IPA-producing bacteria [92]. In contrast to traditional antioxidants, IPA exhibits sustained protective effects because it is a potent hydroxyl radical scavenger resistant to autoxidation [93]. Mechanistically, IPA stabilizes mitochondrial function by preventing damage to mitochondrial DNA, lowering ROS leakage, and preserving membrane potential [94]. Preclinical studies have demonstrated the neuroprotective effects of IPA in models of PD, where it reduces dopaminergic neuronal loss, and AD, where it mitigates oxidative toxicity induced by amyloid-β. These results highlight the direct translational potential of IPA as a promising indole metabolite [95]. Research indicates that gut microbiota-derived IPA contributes to the protection of microglia against inflammation, thereby enhancing neuronal function [96].

### 4.3. Melatonin (An Indoleamine)

Melatonin is a naturally occurring tryptophan-derived hormone that is primarily secreted by the pineal gland during darkness. Melatonin controls several biological functions, including sleep, circadian rhythm, immunity, and reproduction. In addition, melatonin exhibits anti-inflammatory, free-radical-scavenging, and antioxidant properties. It scavenges reactive oxygen and nitrogen species while also boosting endogenous antioxidant defenses, preventing tissue damage, and suppressing proinflammatory cytokine transcriptional factors [97]. As an endogenous indoleamine derived from tryptophan, melatonin functions both as a circadian rhythm regulator and a potent antioxidant [98]. Melatonin metabolism in the CNS is of considerable interest for numerous reasons. Melatonin can enter the brain through the pineal recess or via uptake from the circulation, and certain brain regions have been hypothesized to synthesize it locally. Melatonin has been shown to provide neuroprotection in multiple model systems, and its therapeutic potential has been explored in attempts to prevent neurodegeneration [99]. Melatonin directly scavenges ROS and RNS, such as singlet oxygen, peroxynitrite, and hydroxyl radicals, while also enhancing endogenous antioxidant defenses through up-regulation of catalase, glutathione peroxidase, and superoxide dismutase [100]. In addition to neuroprotection from its free radical-scavenging activity, melatonin exerts pleiotropic effects that impact the immune and cardiovascular systems. According to studies, melatonin plays a role in neuronal survival, proliferation, and differentiation, including axogenesis and dendritogenesis [101]. These effects are comparable to those induced by neurotrophin-3, neurotrophin-4/5, nerve growth factor, and BDNF. Additionally, melatonin exhibits anti-inflammatory and apoptotic effects in certain brain regions, similar to the actions of neurotrophic factors [102]. Since circadian disruption is closely linked to oxidative stress and cognitive decline, its capacity to synchronize circadian rhythms further enhances its neuroprotective potential [103].

### 4.4. Other Emerging Indole-Based Molecules

Many diverse synthetic indole derivatives with enhanced neuroprotective properties have been developed by recent efforts in medicinal chemistry. Molecules with enhanced BBB permeability, metabolic stability, and antioxidant potency have been produced by structural alterations of the indole scaffold [29]. For instance, indole-based hybrids with phenolic or carboxamide substituents exhibit better free radical-scavenging ability than natural indoles. Melatonin and caffeic acid have been hybridized to produce indole-based amide derivatives with augmented antioxidant activity. These substances outperformed Trolox in certain assays and demonstrated better radical-scavenging ability than benzamide analogs [104]. Additionally, some analogs selectively target signaling cascades associated with neuronal survival, including inhibition of glycogen synthase kinase-3β (GSK-3β) and modulation of PI3K/Akt signaling [105]. Preliminary in vitro and in vivo studies reveal that they are effective at reducing oxidative damage and neuroinflammation, and preventing apoptosis in Alzheimer’s and PD models. Collectively, these findings demonstrate the translational potential of rationally designed indole derivatives as next-generation redox modulators for neuroprotection [106].

Overall, indole-derived compounds constitute a broad class of redox modulators with substantial neuroprotective potential. Naturally occurring indoles such as I3C and IPA exhibit potent anti-inflammatory and antioxidant qualities, whereas melatonin uniquely integrates circadian regulation with redox homeostasis [107]. Synthetic indole derivatives broaden this therapeutic landscape by providing optimized pharmacokinetic profiles and increased potency [14]. These molecules target key mechanisms underlying neuronal vulnerability, such as oxidative stress, mitochondrial dysfunction, neuroinflammation, and impaired neurotrophic signaling. Their multimodal actions make indole derivatives compelling candidates for therapeutic development in neurodegenerative disorders where redox dysregulation plays a central pathogenic role.

## 5. Mechanistic Insights into Neuronal Protection

**Figure 5:**
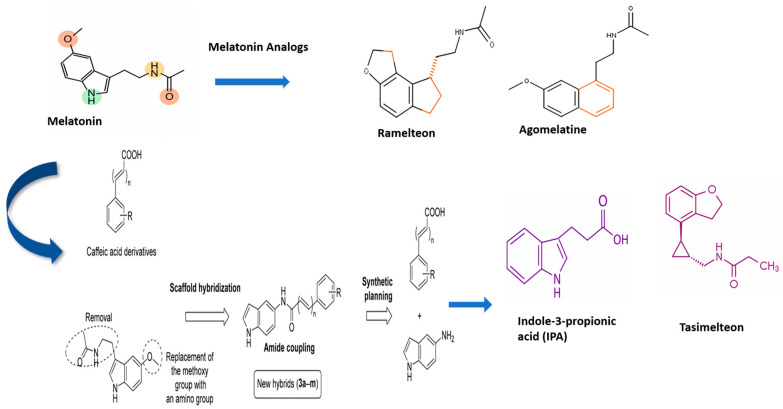
Chemical structures of representative melatonin analogs (ramelteon, agomelatine), the melatonin-caffeic acid hybrid, and clinically investigated indole-derived compounds (Tasimelteon and IPA) to better highlight medicinal chemistry trends and structure-activity relations in the Melatonin analogs. The chemical structure of Ramelteon shows the structural differences from melatonin are highlighted. The chemical structure of Agomelatine shows that the formula’s colored aromatic ring indicates the structural difference when compared to melatonin, which contains an indole group.

**Figure 6:**
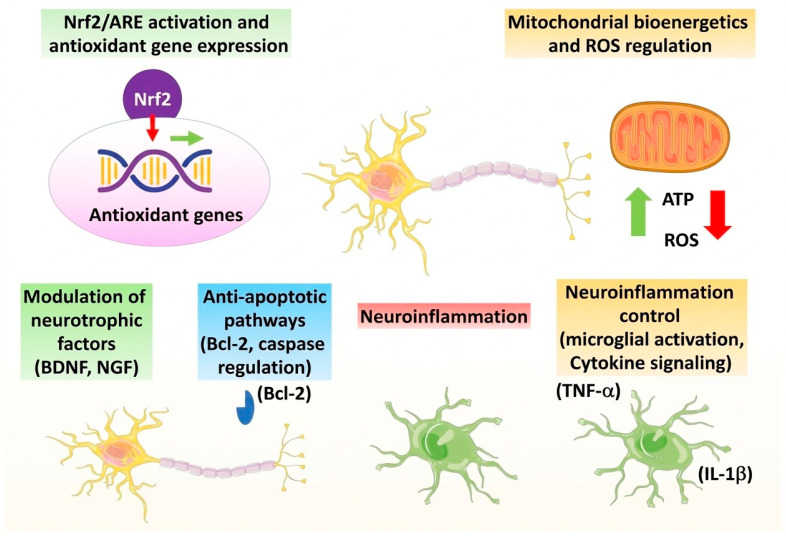
Proposed neuroprotective mechanisms of selected indole-derived compounds. Selected indole-derived compounds, including indole-3-carbinol (I3C), indole-3-propionic acid (IPA), melatonin, and related synthetic indole derivatives, exert neuroprotective effects through multiple interconnected pathways. These include activation of Nrf2/ARE signaling, regulation of mitochondrial bioenergetics and ROS production, modulation of neurotrophic factors (BDNF and NGF), inhibition of apoptotic signaling, and attenuation of neuroinflammation through regulation of microglial activation and cytokine signaling.

Neuronal encompasses several interdependent molecular events, such as restoration of the redox balance, preservation of mitochondrial functions, modulation of neuroinflammation, neurotrophic signaling preservation, and inhibition of apoptosis [108]. In the context of this review, these mechanisms are supposed to be considered compound-specific. It has been reported that certain indole-derived compounds (e.g., indole-3-carbinol, 3,3′-diindolylmethane, indole-3-propionic acid, and melatonin) modulate antioxidant and neuroprotective pathways in some experimental models [23,29,36]. Therefore, rather than stating that all compounds containing indole have neuronal protection, this section describes specific mechanisms that have been highlighted by diverse research. Therefore, this section focuses on representative neuroprotective mechanisms reported for selected indole derivatives rather than implying that all indole-containing compounds exert neuroprotective effects. These integrated mechanisms are summarized in Figure 5. Proposed neuroprotective mechanisms of selected indole-derived compounds, including I3C, IPA, melatonin and related synthetic indole derivatives, exert neuroprotective effects through multiple interconnected pathways (Figure 6).

### 5.1. Nrf2/ARE Activation and Antioxidant Gene Expression

Activation of the Nrf2 (nuclear factor erythroid 2-related factor 2) signaling pathway is a key mechanism through which neurons mitigate oxidative stress [109]. Once activated, Nrf2 translocates to the nucleus and binds to the antioxidant response element (ARE), promoting the transcription of cytoprotective genes, such as heme oxygenase-1 (HO-1), glutathione S-transferase, and NAD(P)H:quinone oxidoreductase 1 (NQO1) [110]. This coordinated response strengthens cellular antioxidant capacity, detoxifies reactive intermediates, and re-establishes redox homeostasis. Indole-derived compounds, particularly I3C, IPA, and melatonin, have been shown to effectively activate Nrf2/ARE signaling, thereby initiating a broad-spectrum antioxidant defense in neuronal systems [43].

### 5.2. Mitochondrial Bioenergetics and ROS Regulation

Mitochondria are both a major source and a primary target of ROS in neurons [111]. Oxidative stress impairs mitochondrial respiratory chain function, lowers ATP production, and activates apoptotic cascades [112]. Several indole compounds can support electron flux through the respiratory chain, preserve mitochondrial membrane potential, and reduce electron leakage, thereby lowering free radical production [113]. Furthermore, because they can participate in single-electron transfer reactions without prooxidant intermediates, some indoles may function as strong antioxidants [114]. In 1999, a study reported that indole-3-propionic acid (IPA), a compound linked to melatonin, may be the most effective naturally occurring hydroxyl radical scavenger, efficiently neutralizing free radicals through electron donation [115]. IPA, for example, preserves mitochondrial DNA integrity and prevents the oxidative disruption of mitochondrial networks, whereas melatonin enhances mitochondrial biogenesis through PGC-1α activation. These actions help sustain neuronal bioenergetics under stress conditions, preventing energy failure and cell death [116].

### 5.3. Modulation of Neurotrophic Factors (BDNF, NGF)

Antioxidant defenses in the mammalian CNS counterbalance the production of ROS. When ROS levels become excessive, endogenous antioxidant systems become overwhelmed, which can lead to oxidative cellular stress. Consequently, ROS are usually considered harmful molecules that oxidize membrane lipids, alter protein structure, damage nucleic acids, and impair synaptic plasticity. High ROS levels are linked to cognitive decline, as observed in certain neurodegenerative disorders and age-related deterioration of neuroplasticity [117]. BDNF and nerve growth factor (NGF) are particularly vulnerable to oxidative and inflammatory insults [118]. Indole-derived compounds enhance neurotrophic support by upregulating BDNF and NGF expression and potentiating their downstream signaling pathways, such as TrkB and PI3K/Akt [119]. Melatonin, for instance, restores BDNF levels in oxidative stress models, thereby improving synaptic function and cognitive outcomes [120]. This modulation of neurotrophic pathways provides an important link between antioxidant effects and functional neuroprotection.

### 5.4. Antiapoptotic Pathways (Bcl-2, Caspase Regulation)

Oxidative stress and mitochondrial dysfunction both activate intrinsic apoptotic pathways, causing cytochrome c release, caspase activation, and downregulation of antiapoptotic proteins [121]. Indole-derived compounds attenuate apoptosis by upregulating Bcl-2, an antiapoptotic protein, and inhibiting proapoptotic factors such as Bax and cleaved caspase-3 [122]. By maintaining mitochondrial membrane stability and preventing cytochrome c leakage, these compounds effectively block the execution phase of apoptosis, preserving neuronal viability under stress conditions [123]. The Bcl-2 family plays a unique role in regulating neuronal cell survival because it controls both caspase-dependent and caspase-independent cell death pathways. Studies employing targeted gene knockouts of specific Bcl-2 family members, as well as transgenic mouse models overexpressing either antiapoptotic or proapoptotic Bcl-2 family members, have substantiated their significance in the nervous system. Evidence from human brain tissue and experimental animal models of neuropathological disorders further validates the hypothesis that the Bcl-2 family modulates cell death in the mature nervous system. Furthermore, pharmacological intervention targeting Bcl-2 family activity may offer therapeutic benefit in human neurological disorders, including stroke and NDs [124].

### 5.5. Neuroinflammation Control (Microglial Activation, Cytokines)

Chronic microglial activation and the release of proinflammatory cytokines, including TNF-α, IL-1β, and IL-6, amplify oxidative stress and neuronal damage [125]. Indole-derived compounds exert strong immunomodulatory effects by suppressing microglial overactivation and promoting a shift in microglial phenotype from the proinflammatory (M1) to the anti-inflammatory (M2) phenotype [36,126,127]. Additionally, they suppress key inflammatory signaling pathways, such as NF-κB and MAPKs, reducing cytokine production and promoting a neuroprotective milieu [128]. This combined antioxidant and anti-inflammatory action disrupts the self-perpetuating cycle of oxidative stress and inflammation in neurodegeneration. Overall, indole-derived compounds confer multifaceted neuroprotection by coordinating a network of antioxidant, antiapoptotic, neurotrophic, and anti-inflammatory mechanisms. Through Nrf2/ARE activation, preservation of mitochondrial function, enhancement of BDNF and NGF signaling, inhibition of apoptotic cascades, and regulation of microglial responses, these compounds effectively offset the detrimental effects of oxidative stress and neuroinflammation. Collectively, these mechanistic insights underscore the therapeutic potential of indole derivatives as promising agents for preserving neuronal integrity and attenuating neurodegenerative processes.

## 6. Translational and Clinical Perspectives

This section reviews the translational potential of selected indole-derived compounds in neurodegenerative diseases, with emphasis on the progression from preclinical evidence to clinical investigation. While compounds such as melatonin have advanced to human studies, many other indole derivatives, including indole-3-propionic acid (IPA), indole-3-carbinol (I3C), and 3,3′-diindolylmethane (DIM), remain largely supported by preclinical data. Therefore, understanding the opportunities and limitations associated with their clinical translation is essential for the future development of indole-based therapeutics.

### 6.1. Preclinical Evidence in Neurodegenerative Models

Indole-derived compounds have demonstrated strong neuroprotective effects across diverse preclinical models of neurodegenerative disorders, including AD, PD, ALS, and ischemic stroke [129]. In these models, indoles reduce oxidative stress, stabilize mitochondrial function, augment neurotrophic signaling, and suppress neuroinflammation, leading to improved neuronal survival, synaptic integrity, and behavioral outcomes [130,131]. Robust neuroprotective effects have been demonstrated for melatonin, IPA, I3C, and DIM in cellular and animal models of AD, PD, and other neurodegenerative disorders.

### 6.2. Human Clinical Trial

Although clinical evidence remains limited, recent findings support the translational relevance of indole compounds. Melatonin supplementation has been reported to boost sleep quality [132], cognitive performance [133], and antioxidant status in older adults and patients with neurodegenerative conditions [133]. A summary of representative clinical trials investigating indole-derived compounds in neurological disorders is provided in Table 2. Additionally, in a 12-week randomized, double-blind, placebo-controlled, multicenter clinical trial involving healthy elderly participants, probiotic supplementation with Bifidobacterium bifidum BGN4 and Bifidobacterium longum BORI significantly modulated tryptophan metabolism and increased circulating gut microbiota-derived indole-3-propionic acid (IPA). In vitro tests validated IPA’s mechanistic significance by demonstrating decreased microglial inflammatory responses and better neuronal-supportive signaling. Although this study did not directly evaluate IPA supplementation or enroll individuals with neurodegenerative diseases, it does provide important human clinical evidence associating microbiota-derived IPA with neuroprotective pathways in aging [96]. These findings suggest that indoles may confer protective effects in humans consistent with those observed in preclinical models.

### 6.3. Limitations and Challenges

Despite promising results in preclinical studies, several challenges still limit the clinical development of indole-derived compounds for neurodegenerative diseases. One major challenge is that different indole compounds vary in their ability to cross the blood–brain barrier and reach the brain at therapeutically effective concentrations. In addition, factors such as bioavailability, metabolism, and stability can influence their effectiveness. For many compounds, the optimal dose and long-term safety profile have not yet been fully established. Another limitation is the lack of reliable biomarkers to assess treatment responses in patients. Moreover, although melatonin has been investigated in clinical studies, most indole-derived compounds, including indole-3-propionic acid (IPA), indole-3-carbinol (I3C), and 3,3′-diindolylmethane (DIM), have primarily been evaluated in experimental models. Therefore, further clinical studies are needed to confirm their safety and therapeutic potential in neurodegenerative diseases.

### 6.4. Opportunities for Therapeutic Development

Innovative strategies are being investigated to overcome these translational barriers. Nanoparticle-based delivery systems can enhance brain uptake, stability, and controlled release of indole compounds [134]. Future studies should focus on improving the therapeutic potential of indole-derived compounds by enhancing their biological activity, stability, and ability to cross the blood–brain barrier. The development of advanced drug-delivery systems, such as nanoparticles and liposomes, may improve drug delivery to the brain and increase therapeutic efficacy. Combination therapies involving indole derivatives and existing neuroprotective agents may also provide greater benefits by targeting multiple disease mechanisms simultaneously. In addition, the identification of reliable biomarkers and the application of personalized treatment strategies could support the development of more effective indole-based therapies. Together, these approaches may help translate promising preclinical findings into successful clinical applications for neurodegenerative diseases.

## 7. Future Directions

Future research should focus on determining the exact structure-activity interactions of indole-derived compounds in order to identify chemical changes that improve antioxidant potency, Nrf2/ARE activation, neurotrophic signaling, and blood–brain barrier permeability. Microbiota-derived indoles, such as indole-3-propionic acid (IPA), should be studied in depth to understand the processes that relate gut microbial metabolism to neuronal redox homeostasis and neuroprotection. Comparative research on individual indole derivatives’ effects on Nrf2/ARE, BDNF-TrkB, PI3K/Akt, and NF-κB signaling pathways is required to discover interesting therapeutic options. Advanced delivery technologies, such as nanoparticle and lipid-based formulations, should be developed to increase CNS bioavailability and therapeutic efficacy.

Emerging data support the function of gut-derived indole metabolites, particularly IPA, in regulating brain health via the gut–brain axis. Future research should focus on identifying the microbial taxa that produce indole and developing techniques to boost endogenous production of neuroprotective indole metabolites. Personalized dietary strategies and micro-biome-targeted interventions could harness these metabolites to optimize endogenous neuroprotection, supporting the development of precision nutrition strategies in neurodegenerative disorders.

To translate preclinical promise into clinical benefit, well-designed trial designs are essential. Key considerations include optimizing the dosage, timing, and formulation of indole compounds, selecting sensitive biomarkers for oxidative stress and neurotrophic activity, and identifying appropriate patient populations. Incorporating these parameters will improve the likelihood of demonstrating clinical efficacy and establishing in-dole-based therapeutics as viable treatment options. Future research integrating redox modulation, neurotrophic support, and microbiome-derived indole metabolites, combined with rational clinical trial designs, offers strong potential for developing personalized, indole-based neuroprotective strategies. Such approaches may advance the prevention and treatment of neurodegenerative disorders by translating mechanistic insights into practical therapy.

## 8. Conclusions

Indole derivatives are versatile neuroprotective agents that act through multiple redox-modulating mechanisms, including activation of Nrf2/ARE signaling, stabilization of mitochondrial function, enhancement of neurotrophic pathways, inhibition of apoptosis, and attenuation of neuroinflammation. These mechanisms collectively preserve neuronal integrity and function, underscoring the therapeutic promise of indole-based strategies in NDs. Further progress in mechanistic understanding, bioavailability optimization, personalized nutrition, and targeted clinical translation will be essential to fully realize the potential of antioxidant-based neuroprotective strategies in the future.

The neuroprotective potential of indole-based compounds arises from their multitargeted activity, including redox modulation, neurotrophic effects, and anti-inflammatory properties. Optimized delivery systems and personalized strategies to exploit these mechanisms hold strong promise for next-generation antioxidant-based therapies in neurodegenerative disorders. 

## Figures and Tables

**Table 1:** Neuroprotective Roles of Indole-Derived Compounds.

Compound	Source	Key Mechanisms	Relevance in Neurodegeneration	Ref.
I3C	Glucobrassicin	Activation of SIRT1/AMPK pathway.	rotenone (ROT)-induced PD in male albino rats	[79]
I3C	Glucobrassicin	Nrf2 signaling activation, antioxidant enzyme regulation, and chaperone-mediated proteostasis enhancement	Cerebral ischemia/reperfusion injury rat model	[80]
Indole-3-propionic acid (IPA)	Gut microbiota-derived metabolite of tryptophan; also present in plant-based foods (fruits, vegetables)	Strong antioxidant (no pro-oxidant activity) Scavenging free radicals, which prevents peroxy-radicals from forming AhR and PXR signaling pathway activation Effects that mitigate inflammation Alterations in the gut–brain axis The impact of cytostatic agents on cancerous cells	Neuroprotection in Alzheimer’s (AD), Parkinson’s (PD), and stroke models. Minimizes oxidative stress and neuronal loss	[27]
Indole-based SIRT3 modulators (IMFW-1, IMTW-5, IM24DCW-16)	Synthetic	SIRT3 activation, ROS reduction, antioxidant enzyme upregulation, and mitochondrial protection.	Neuroprotection in Parkinson’s disease by reducing oxidative stress and supporting mitochondrial function.	
Indole-3-propionic acid (IPA)	Microbiota-derived indoles	Inhibition of amyloid aggregation Modulation of host–microbiota–brain axis	Delays onset and progression of Alzheimer’s disease (AD)	[81]
Melatonin analogs	Synthetic (melatonin-derived)	Antioxidant, anti-amyloid, ROS reduction	AD neuroprotection	[82]
3,3′-Diindolylmethane (DIM)	Derived from dietary indole-3-carbinol (I3C) found in cruciferous vegetables; produced under acidic conditions in the stomach	Antiapoptotic, anti-autophagic, AhR modulation, HDAC activation	Ischemia-induced neuroprotection	[83]
DIM (bioactive metabolite)	Synthetic (DIM-based)	Anti-inflammatory, neuroprotective, and blood–brain barrier penetrant Neuroprotection via preservation of dopaminergic neurons	Prevents dopaminergic neuron loss in PD	[84]
Hydroxyindoles (3HI, 4HI)	Natural/synthetic indole derivatives	Anti-amyloid (inhibits Aβ aggregation via aromatic interaction disruption)	AD neuroprotection	[85]

**Table 2:** Overview of Clinical Trials Evaluating Indole-Derived Compounds and Related Molecules in Neurological and Neuropsychiatric Disorders.

Compound Name	Study Title	Condition	Status	NCT No.	Intervention/Treatment	Doses
Melatonin	Effect of Melatonin and Transcranial Direct Current Stimulation (tDCS) on Neuroplasticity and the Heat-pain Detection Threshold in Healthy Subjects: Randomized, Double-blind, Crossover Trial	Change from baseline Brain-Derived Neurotrophic Factor (BDNF)	Not updated	NCT02195271	Melatonin + tDCS	0.25 mg/Kg sl before tDCS tDCS: Transcranial direct current stimulation one time. Dose 2 mA, 20 s.
indole-3-propionic acid (IPA)	Indole-3-PROpionic Acid Clinical Trials—a Pilot Study (iPROACT-pilot)	Brain-derived neurotrophic factor measured in plasma samples using ELISA or mesoscale.	completed	NCT06674018	Placebo	50 mg IPA or 120 mg IPA or 500 mg IPA or placebo every morning for 14 days.
Indoximod	A Phase I Trial of Indoximod and Temozolomide-Based Therapy for Children With Progressive Primary Brain Tumors	The goal of this pediatric study is to bring IDO-based immunotherapy into the clinic for children with brain tumors. This study will provide a foundation for future pediatric trials testing indoximod combined with radiation and temozolomide in the up-front setting for patients with newly diagnosed central nervous system tumors.	completed	NCT02502708	administered orally twice daily.	Initial dosing will be 12.8 mg/kg/dose BID with escalation planned to 22.4 mg/kg/dose BID.
Indoximod	Genetic and Biochemical Markers of Interferon-Induced Depression.	Depression	completed	NCT00252538	Cohort	Non-Probability Sample
Tryptamine	Melatonin for Huntington’s Disease (HD) Gene Carriers With HD-Related Sleep Disturbance—a Pilot Study	Huntington Disease	completed	NCT04421339	Dietary Supplement: Melatonin Other: Placebo	Participants will be administered melatonin 5 mg once a day (30 min prior to bedtime) for four weeks, followed by one-week washout before crossing-over.

## Data Availability

No new data were created or analyzed in this study. Data sharing is not applicable to this article.

## Abbreviations

The following abbreviations are used in this manuscript:

AD	Alzheimer’s Disease
AhR	Aryl Hydrocarbon Receptor
AHDs	Antiherpetic Drugs
ALS	Amyotrophic Lateral Sclerosis
AMPK	AMP-Activated Protein Kinase
APO	Apoptosis
ARE	Antioxidant Response Element
ATP	Adenosine Triphosphate
Aβ	Amyloid-beta
BBB	Blood–Brain Barrier
Bcl-2	B-cell lymphoma 2
BDNF	Brain-Derived Neurotrophic Factor
BID	Twice Daily
Ca2+	Calcium Ion
CK1	Casein Kinase 1
CNS	Central Nervous System
COX-2	Cyclooxygenase-2
CS	Chitosan
DIM	3,3′-Diindolylmethane
DNA	Deoxyribonucleic Acid
ELISA	Enzyme-Linked Immunosorbent Assay
ER	Endoplasmic Reticulum
FDA	Food and Drug Administration
GPR30	G Protein-Coupled Estrogen Receptor 30
GPx	Glutathione Peroxidase
GSH	Glutathione
GSK-3β	Glycogen Synthase Kinase-3 Beta
HD	Huntington’s Disease
HDAC	Histone Deacetylase
HNE	4-Hydroxy-2-Nonenal
HO-1	Heme Oxygenase-1
I3C	Indole-3-Carbinol
IDO	Indoleamine 2,3-Dioxygenase
IL-1β	Interleukin-1 Beta
IL-6	Interleukin-6
IL-10	Interleukin-10
IPA	Indole-3-Propionic Acid
LPO	Lipid Peroxidation
LRRK2	Leucine-Rich Repeat Kinase 2
LTD	Long-Term Depression
MAP2	Microtubule-Associated Protein 2
MAPKs	Mitogen-Activated Protein Kinases
mA	Milliampere
mg	Milligram
NCT	National Clinical Trial Number
NDs	Neurodegenerative Diseases
NF-κB	Nuclear Factor Kappa B
NGF	Nerve Growth Factor
NQO1	NAD(P)H Quinone Oxidoreductase 1
Nrf2	Nuclear Factor Erythroid 2-Related Factor 2
ONOO−	Peroxynitrite
PBPK	Physiologically Based Pharmacokinetic
PD	Parkinson’s Disease
PGC-1α	Peroxisome Proliferator-Activated Receptor Gamma Coactivator 1-alpha
PI3K/Akt	Phosphoinositide 3-Kinase/Protein Kinase B
PSD	Postsynaptic Density
PSD-95	Postsynaptic Density Protein 95
PUFAs	Polyunsaturated Fatty Acids
PXR	Pregnane X Receptor
RNS	Reactive Nitrogen Species
ROS	Reactive Oxygen Species
ROT	Rotenone
SCA	Spinocerebellar Ataxia
SIRT1	Sirtuin 1
SIRT3	Sirtuin 3
Sl	Sublingual
SOD1	Superoxide Dismutase 1
SYP	Synaptophysin
tDCS	Transcranial Direct Current Stimulation
TNF-α	Tumor Necrosis Factor-alpha
Trk	Tropomyosin Receptor Kinase
TrkB	Tropomyosin Receptor Kinase B
Trp	Tryptophan
UPR	Unfolded Protein Response

## References

[1] Pathak N., Vimal S.K., Tandon I., Agrawal L., Hongyi C., Bhattacharyya S. (2022). Neurodegenerative disorders of alzheimer, parkinsonism, amyotrophic lateral sclerosis and multiple sclerosis: An early diagnostic approach for precision treatment. Metab. Brain Dis..

[2] Cenini G., Lloret A., Cascella R. (2019). Oxidative stress in neurodegenerative diseases: From a mitochondrial point of view. Oxidative Med. Cell. Longev..

[3] Bellanti F., Coda A.R.D., Trecca M.I., Lo Buglio A., Serviddio G., Vendemiale G. (2025). Redox imbalance in inflammation: The interplay of oxidative and reductive stress. Antioxidants.

[4] Pizzino G., Irrera N., Cucinotta M., Pallio G., Mannino F., Arcoraci V., Squadrito F., Altavilla D., Bitto A. (2017). Oxidative stress: Harms and benefits for human health. Oxidative Med. Cell. Longev..

[5] Wang X., Dong B., Gan Q., Li J., Wu P., Guan Y., Wang J. (2025). Unraveling the Vicious Cycle: Oxidative Stress and Neurotoxicity in Neurodegenerative Diseases. FASEB BioAdvances.

[6] Liu J., Han X., Zhang T., Tian K., Li Z., Luo F. (2023). Reactive oxygen species (ROS) scavenging biomaterials for anti-inflammatory diseases: From mechanism to therapy. J. Hematol. Oncol..

[7] He L., He T., Farrar S., Ji L., Liu T., Ma X. (2017). Antioxidants maintain cellular redox homeostasis by elimination of reactive oxygen species. Cell. Physiol. Biochem..

[8] Ampofo E., Lachnitt N., Rudzitis-Auth J., Schmitt B.M., Menger M.D., Laschke M.W. (2017). Indole-3-carbinol is a potent inhibitor of ischemia–reperfusion–induced inflammation. J. Surg. Res..

[9] Wei P.-C., Lee-Chen G.-J., Chen C.-M., Wu Y.-R., Chen Y.-J., Lin J.-L., Lo Y.-S., Yao C.-F., Chang K.-H. (2019). Neuroprotection of Indole-Derivative Compound NC001-8 by the Regulation of the NRF2 Pathway in Parkinson’s Disease Cell Models. Oxidative Med. Cell. Longev..

[10] Ciaglia T., Miranda M.R., Di Micco S., Vietri M., Smaldone G., Musella S., Di Sarno V., Auriemma G., Sardo C., Moltedo O. (2024). Neuroprotective potential of indole-based compounds: A biochemical study on antioxidant properties and amyloid disaggregation in neuroblastoma cells. Antioxidants.

[11] Sreejith P., Beyo R., Divya L., Vijayasree A., Manju M., Oommen O. (2007). Triiodothyronine and melatonin influence antioxidant defense mechanism in a teleost Anabas testudineus (Bloch): In vitro study. Indian J. Biochem. Biophys..

[12] Guerrero J.M., Reiter R.J. (2002). Melatonin-immune system relationships. Curr. Top. Med. Chem..

[13] Mohareb R.M., Ahmed H.H., Elmegeed G.A., Abd-Elhalim M.M., Shafic R.W. (2011). Development of new indole-derived neuroprotective agents. Bioorganic Med. Chem..

[14] Porwal A., Rajendiran A., Alam P., Singh H., Singh K., Dubey A. (2024). Indole Moiety in Organic Synthesis: A Comprehensive Review of Methods and Mechanisms. Int. J. Pharm. Investig..

[15] Yu B., Li N., Fu C. (2023). Privileged Scaffolds in Drug Discovery.

[16] Mayer S., Keglevich P., Keglevich A., Hazai L. (2021). New anticancer vinca alkaloids in the last decade-A mini-review. Curr. Org. Chem..

[17] Hasan N.K., El-Khouly E.A., Mahmoud Z., Kandeel M.M. (2025). A Deep Insight into the Indole Nucleus: Pharmacological Action, Structure-Activity Relationship, and Eco-Friendly Synthetic Approaches. Curr. Org. Chem..

[18] de Sa Alves F.R., Barreiro E.J., Manssour Fraga C.A. (2009). From nature to drug discovery: The indole scaffold as a ‘privileged structure’. Mini Rev. Med. Chem..

[19] Silva Moratório de Moraes R., Mestre Botelho A.B., Tavares de Almeida Pinto G., Couto Rodrigues S., Miranda Martins M.T., Alves Soares D.L., Cardoso Cruz C., de Almeida Pinto A., Rodrigues Fintelman Dias F., Dias Fernandes P. (2025). Chemistry, Applications, and Synthesis Methods of Indole Derivatives: A Comprehensive Review. Chem. Rec..

[20] Di Martino R.M.C. (2016). Naturally Inspired Privileged Structures in Drug Discovery: Multifunctional Compounds for Alzheimer’s Disease Treatment. Ph.D. Thesis.

[21] Padmapriya M., Hakkimane S.S., Gaonkar S.L. (2025). Synthetic approaches, emerging applications, and challenges of indole-based five-membered heterocycles in medicinal chemistry. Discov. Appl. Sci..

[22] Crabtree G.W., Gogos J.A. (2018). Role of endogenous metabolite alterations in neuropsychiatric disease. ACS Chem. Neurosci..

[23] Holeček M. (2026). Serotonin, Kynurenine, and Indole Pathways of Tryptophan Metabolism in Humans in Health and Disease. Nutrients.

[24] Korshunov K.S., Blakemore L.J., Trombley P.Q. (2017). Dopamine: A modulator of circadian rhythms in the central nervous system. Front. Cell. Neurosci..

[25] Huang Y., Zhao M., Chen X., Zhang R., Le A., Hong M., Zhang Y., Jia L., Zang W., Jiang C. (2023). Tryptophan metabolism in central nervous system diseases: Pathophysiology and potential therapeutic strategies. Aging Dis..

[26] Shaw C., Hess M., Weimer B.C. (2023). Microbial-derived tryptophan metabolites and their role in neurological disease: Anthranilic acid and anthranilic acid derivatives. Microorganisms.

[27] Owe-Larsson M., Drobek D., Iwaniak P., Kloc R., Urbanska E.M., Chwil M. (2025). Microbiota-derived tryptophan metabolite indole-3-propionic acid-emerging role in neuroprotection. Molecules.

[28] Yin J., Zhang Y., Liu X., Li W., Hu Y., Zhang B., Wang S. (2023). Gut microbiota-derived indole derivatives alleviate neurodegeneration in aging through activating GPR30/AMPK/SIRT1 pathway. Mol. Nutr. Food Res..

[29] Drăgoi C.M., Nicolae A.-C., Dumitrescu I.-B. (2026). The Indole Scaffold in Biochemistry and Therapeutics: A Privileged Structure with Diverse Chemical, Biological, and Clinical Significance. Targets.

[30] Fan L., Zhu X., Liu X., He F., Yang G., Xu C., Yang X. (2023). Recent Advances in the Synthesis of 3, n-Fused Tricyclic Indole Skeletons via Palladium-Catalyzed Domino Reactions. Molecules.

[31] Bowman C., Denis M., Canesi S. (2025). Recent strategy for the synthesis of indole and indoline skeletons in natural products. Chem. Commun..

[32] Yang H.-M. (2025). Overcoming the Blood–Brain Barrier: Advanced Strategies in Targeted Drug Delivery for Neurodegenerative Diseases. Pharmaceutics.

[33] Kafi-De St Hilaire S., Merica H., Gaillard J.-M. (1984). The effects of indalpine—A selective inhibitor of 5-HT uptake-—On rat paradoxical sleep. Eur. J. Pharmacol..

[34] Xue H., Chen X., Yu C., Deng Y., Zhang Y., Chen S., Chen X., Chen K., Yang Y., Ling W. (2022). Gut microbially produced indole-3-propionic acid inhibits atherosclerosis by promoting reverse cholesterol transport and its deficiency is causally related to atherosclerotic cardiovascular disease. Circ. Res..

[35] Fathi M., Vakili K., Yaghoobpoor S., Tavasol A., Jazi K., Hajibeygi R., Shool S., Sodeifian F., Klegeris A., McElhinney A. (2022). Dynamic changes in metabolites of the kynurenine pathway in Alzheimer’s disease, Parkinson’s disease, and Huntington’s disease: A systematic Review and meta-analysis. Front. Immunol..

[36] Barresi E., Baglini E., Poggetti V., Castagnoli J., Giorgini D., Salerno S., Taliani S., Da Settimo F. (2024). Indole-based compounds in the development of anti-neurodegenerative agents. Molecules.

[37] Ball K., Bouzom F., Scherrmann J.-M., Walther B., Declèves X. (2013). Physiologically based pharmacokinetic modelling of drug penetration across the blood–brain barrier—Towards a mechanistic IVIVE-based approach. AAPS J..

[38] Vellingiri D. (2025). Indole derivatives in smart polymeric formulations for targeted management of neurodegenerative disorders. J. Carcinog..

[39] Hitchcock S.A., Pennington L.D. (2006). Structure-brain exposure relationships. J. Med. Chem..

[40] Wang Z., Hu J., Yang X., Feng X., Li X., Huang L., Chan A.S. (2018). Design, synthesis, and evaluation of orally bioavailable quinoline–indole derivatives as innovative multitarget-directed ligands: Promotion of cell proliferation in the adult murine Hippocampus for the treatment of alzheimer’s disease. J. Med. Chem..

[41] Zeng W., Han C., Mohammed S., Li S., Song Y., Sun F., Du Y. (2024). Indole-containing pharmaceuticals: Targets, pharmacological activities, and SAR studies. RSC Med. Chem..

[42] Omar F., Tareq A.M., Alqahtani A.M., Dhama K., Sayeed M.A., Emran T.B., Simal-Gandara J. (2021). Plant-based indole alkaloids: A comprehensive overview from a pharmacological perspective. Molecules.

[43] Singh A.A., Yadav D., Khan F., Song M. (2024). Indole-3-Carbinol and its derivatives as neuroprotective modulators. Brain Sci..

[44] Schütz B., Krause F.F., Taudte R.V., Zaiss M.M., Luu M., Visekruna A. (2025). Modulation of host immunity by Microbiome-Derived Indole-3-Propionic acid and other bacterial metabolites. Eur. J. Immunol..

[45] Gupta H.K., Jangra J., Ramesh V.K., Mahindru I., Kumar R. (2026). Medicinal chemistry strategies to breach the blood–brain barrier: Structural design principles for brain-targeted therapeutics. Drug Discov. Today.

[46] Mo X., Rao D.P., Kaur K., Hassan R., Abdel-Samea A.S., Farhan S.M., Bräse S., Hashem H. (2024). Indole Derivatives: A Versatile Scaffold in Modern Drug Discovery-An Updated Review on Their Multifaceted Therapeutic Applications (2020–2024). Molecules.

[47] Pietrzak A., Dąbrówka B., Popiół J., Pękala E., Słoczyńska K. (2026). Phase II metabolism in xenobiotic biotransformation: General mechanisms and the underestimated role of microbial systems. Drug Metab. Rev..

[48] Kamata H., Hirata H. (1999). Redox regulation of cellular signalling. Cell. Signal..

[49] Maity-Kumar G., Thal D.R., Baumann B., Scharffetter-Kochanek K., Wirth T. (2015). Neuronal redox imbalance results in altered energy homeostasis and early postnatal lethality. FASEB J..

[50] Nicholls D.G. (2008). Oxidative stress and energy crises in neuronal dysfunction. Ann. N. Y. Acad. Sci..

[51] Angelova P.R., Abramov A.Y. (2016). Functional role of mitochondrial reactive oxygen species in physiology. Free Radic. Biol. Med..

[52] Andrés C.M.C., Pérez de la Lastra J.M., Juan C.A., Plou F.J., Pérez-Lebeña E. (2022). The role of reactive species on innate immunity. Vaccines.

[53] Wang J.-Y., Wen L.-L., Huang Y.-N., Chen Y.-T., Ku M.-C. (2006). Dual effects of antioxidants in neurodegeneration: Direct neuroprotection against oxidative stress and indirect protection via suppression of gliamediated inflammation. Curr. Pharm. Des..

[54] Bogdanov M., Brown R.H., Matson W., Smart R., Hayden D., O’Donnell H., Beal M.F., Cudkowicz M. (2000). Increased oxidative damage to DNA in ALS patients. Free Radic. Biol. Med..

[55] Tabassum N., Kheya I.S., Asaduzzaman S., Maniha S., Fayz A.H., Zakaria A., Noor R. (2020). A review on the possible leakage of electrons through the electron transport chain within mitochondria. Life Sci..

[56] Graeber M.B., Grasbon-Frodl E., Eitzen U.V., Kösel S. (1998). Neurodegeneration and aging: Role of the second genome. J. Neurosci. Res..

[57] Zimmermann K.C., Bonzon C., Green D.R. (2001). The machinery of programmed cell death. Pharmacol. Ther..

[58] Nagley P., Higgins G.C., Atkin J.D., Beart P.M. (2010). Multifaceted deaths orchestrated by mitochondria in neurones. Biochim. Biophys. Acta (BBA)-Mol. Basis Dis..

[59] Schröder M. (2008). Endoplasmic reticulum stress responses. Cell. Mol. Life Sci..

[60] Ong G., Logue S.E. (2023). Unfolding the interactions between endoplasmic reticulum stress and oxidative stress. Antioxidants.

[61] Taso O.V., Philippou A., Moustogiannis A., Zevolis E., Koutsilieris M. (2019). Lipid peroxidation products and their role in neurodegenerative diseases. Ann. Res. Hosp..

[62] Sultana R., Perluigi M., Butterfield D.A. (2013). Lipid peroxidation triggers neurodegeneration: A redox proteomics view into the Alzheimer disease brain. Free Radic. Biol. Med..

[63] Shichiri M. (2014). The role of lipid peroxidation in neurological disorders. J. Clin. Biochem. Nutr..

[64] Perluigi M., Coccia R., Butterfield D.A. (2012). 4-Hydroxy-2-nonenal, a reactive product of lipid peroxidation, and neurodegenerative diseases: A toxic combination illuminated by redox proteomics studies. Antioxid. Redox Signal..

[65] Reed T.T. (2011). Lipid peroxidation and neurodegenerative disease. Free Radic. Biol. Med..

[66] Hidalgo C., Carrasco M.A., Muñoz P., Núñez M.T. (2007). A role for reactive oxygen/nitrogen species and iron on neuronal synaptic plasticity. Antioxid. Redox Signal..

[67] Kumar A., Yegla B., Foster T.C. (2018). Redox signaling in neurotransmission and cognition during aging. Antioxid. Redox Signal..

[68] Kamat P.K., Kalani A., Rai S., Swarnkar S., Tota S., Nath C., Tyagi N. (2016). Mechanism of oxidative stress and synapse dysfunction in the pathogenesis of Alzheimer’s disease: Understanding the therapeutics strategies. Mol. Neurobiol..

[69] Agostinho P., A. Cunha R., Oliveira C. (2010). Neuroinflammation, oxidative stress and the pathogenesis of Alzheimer’s disease. Curr. Pharm. Des..

[70] Bambico F., Bregman T., Diwan M., Li J., Darvish-Ghane S., Li Z., Laver B., Amorim B., Covolan L., Nobrega J. (2015). Neuroplasticity-dependent and-independent mechanisms of chronic deep brain stimulation in stressed rats. Transl. Psychiatry.

[71] Annunziato L., Amoroso S., Pannaccione A., Cataldi M., Pignataro G., D’Alessio A., Sirabella R., Secondo A., Sibaud L., Di Renzo G. (2003). Apoptosis induced in neuronal cells by oxidative stress: Role played by caspases and intracellular calcium ions. Toxicol. Lett..

[72] Mattson M.P., LaFerla F.M., Chan S.L., Leissring M.A., Shepel P.N., Geiger J.D. (2000). Calcium signaling in the ER: Its role in neuronal plasticity and neurodegenerative disorders. Trends Neurosci..

[73] Merighi A., Lossi L. (2022). Endoplasmic reticulum stress signaling and neuronal cell death. Int. J. Mol. Sci..

[74] Li J., Lee B., Lee A.S. (2006). Endoplasmic reticulum stress-induced apoptosis: Multiple pathways and activation of p53-up-regulated modulator of apoptosis (PUMA) and NOXA by p53. J. Biol. Chem..

[75] Logue S.E., Cleary P., Saveljeva S., Samali A. (2013). New directions in ER stress-induced cell death. Apoptosis.

[76] Naim M., Alam O., Alam J., Bano F., Alam P., Shrivastava N. (2016). Recent review on indole: A privileged scaffold structure. Int. J. Pharm. Sci. Res..

[77] Taber D.F., Tirunahari P.K. (2011). Indole synthesis: A review and proposed classification. Tetrahedron.

[78] Zhang M.-Z., Chen Q., Yang G.-F. (2015). A review on recent developments of indole-containing antiviral agents. Eur. J. Med. Chem..

[79] Mohamad K.A., El-Naga R.N., Wahdan S.A. (2022). Neuroprotective effects of indole-3-carbinol on the rotenone rat model of Parkinson’s disease: Impact of the SIRT1-AMPK signaling pathway. Toxicol. Appl. Pharmacol..

[80] Kryl’skii E.D., Popova T.N., Lavrushchev A.I., Popov S.S., Pyatigorskaya N.V. (2025). Indole-3-carbinol exerts neuroprotective effect in cerebral ischaemia/reperfusion through the modulation of Nrf2-mediated antioxidant responses and the restoration of chaperone activity. Arch. Biochem. Biophys..

[81] Pappolla M.A., Perry G., Fang X., Zagorski M., Sambamurti K., Poeggeler B. (2021). Indoles as essential mediators in the gut-brain axis. Their role in Alzheimer’s disease. Neurobiol. Dis..

[82] Gurer-Orhan H., Karaaslan C., Ozcan S., Firuzi O., Tavakkoli M., Saso L., Suzen S. (2016). Novel indole-based melatonin analogues: Evaluation of antioxidant activity and protective effect against amyloid β-induced damage. Bioorganic Med. Chem..

[83] Rzemieniec J., Wnuk A., Lasoń W., Bilecki W., Kajta M. (2019). The neuroprotective action of 3, 3′-diindolylmethane against ischemia involves an inhibition of apoptosis and autophagy that depends on HDAC and AhR/CYP1A1 but not ERα/CYP19A1 signaling. Apoptosis.

[84] De Miranda B.R., Miller J.A., Hansen R.J., Lunghofer P.J., Safe S., Gustafson D.L., Colagiovanni D., Tjalkens R.B. (2013). Neuroprotective efficacy and pharmacokinetic behavior of novel anti-inflammatory para-phenyl substituted diindolylmethanes in a mouse model of Parkinson’s disease. J. Pharmacol. Exp. Ther..

[85] Cohen T., Frydman-Marom A., Rechter M., Gazit E. (2006). Inhibition of amyloid fibril formation and cytotoxicity by hydroxyindole derivatives. Biochemistry.

[86] Licznerska B., Baer-Dubowska W. (2016). Indole-3-carbinol and its role in chronic diseases. Anti-Inflamm. Nutraceuticals Chronic Dis..

[87] Singh A.A., Patil M.P., Kang M.-J., Niyonizigiye I., Kim G.-D. (2021). Biomedical application of Indole-3-carbinol: A mini-review. Phytochem. Lett..

[88] Chen X.-L., Kunsch C. (2004). Induction of cytoprotective genes through Nrf2/antioxidant response element pathway: A new therapeutic approach for the treatment of inflammatory diseases. Curr. Pharm. Des..

[89] Umesalma S., Sudhandiran G. (2010). Differential inhibitory effects of the polyphenol ellagic acid on inflammatory mediators NF-κB, iNOS, COX-2, TNF-α, and IL-6 in 1, 2-dimethylhydrazine-induced rat colon carcinogenesis. Basic Clin. Pharmacol. Toxicol..

[90] Vinjavarapu L.A., Yadava S., Dontiboina H.R., Chakravarthi G., Kakarla R. (2025). Neuroprotective effects of Indole 3-carbinol against Scopolamine-Induced cognitive and memory impairment in rats: Modulation of oxidative stress, inflammatory and cholinergic pathways. Metab. Brain Dis..

[91] Konopelski P., Mogilnicka I. (2022). Biological effects of indole-3-propionic acid, a gut microbiota-derived metabolite, and its precursor tryptophan in mammals’ health and disease. Int. J. Mol. Sci..

[92] Jiang H., Chen C., Gao J. (2022). Extensive summary of the important roles of indole propionic acid, a gut microbial metabolite in host health and disease. Nutrients.

[93] Teoh E.S. (2016). Galeola to gymadenia. Medicinal Orchids of Asia.

[94] Owumi S.E., Najophe E.S., Otunla M.T. (2022). 3-Indolepropionic acid prevented chlorpyrifos-induced hepatorenal toxicities in rats by improving anti-inflammatory, antioxidant, and pro-apoptotic responses and abating DNA damage. Environ. Sci. Pollut. Res..

[95] Pappolla M.A., Martins R.N., Poeggeler B., Omar R.A., Perry G. (2024). Oxidative stress in Alzheimer’s disease: The shortcomings of antioxidant therapies. J. Alzheimer’s Dis..

[96] Kim C.-S., Jung S., Hwang G.-S., Shin D.-M. (2023). Gut microbiota indole-3-propionic acid mediates neuroprotective effect of probiotic consumption in healthy elderly: A randomized, double-blind, placebo-controlled, multicenter trial and in vitro study. Clin. Nutr..

[97] Hacışevki A., Baba B. (2018). An Overview of Melatonin as an Antioxidant Molecule: A Biochemical Approach.

[98] Tan D.-X., Manchester L.C., Esteban-Zubero E., Zhou Z., Reiter R.J. (2015). Melatonin as a potent and inducible endogenous antioxidant: Synthesis and metabolism. Molecules.

[99] Hardeland R. (2010). Melatonin metabolism in the central nervous system. Curr. Neuropharmacol..

[100] Arnao M.B., Hernández-Ruiz J. (2019). Melatonin and reactive oxygen and nitrogen species: A model for the plant redox network. Melatonin Res..

[101] Sarlak G., Jenwitheesuk A., Chetsawang B., Govitrapong P. (2013). Effects of melatonin on nervous system aging: Neurogenesis and neurodegeneration. J. Pharmacol. Sci..

[102] Miranda-Riestra A., Estrada-Reyes R., Torres-Sanchez E.D., Carreño-García S., Ortiz G.G., Benítez-King G. (2022). Melatonin: A neurotrophic factor?. Molecules.

[103] Baser K.H.C., Haskologlu I.C., Erdag E. (2025). Molecular links between circadian rhythm disruption, melatonin, and neurodegenerative diseases: An updated review. Molecules.

[104] Elkamhawy A., Oh N.K., Gouda N.A., Abdellattif M.H., Alshammari S.O., Abourehab M.A., Alshammari Q.A., Belal A., Kim M., Al-Karmalawy A.A. (2023). Novel hybrid indole-based caffeic acid amide derivatives as potent free radical scavenging agents: Rational design, synthesis, spectroscopic characterization, in silico and in vitro investigations. Metabolites.

[105] Alhassan H.H., Janiyani K., Surti M., Adnan M., Patel M. (2025). The dual role of glycogen synthase kinase-3 beta (GSK3β) in neurodegenerative pathologies: Interplay between autophagy and disease progression. Front. Pharmacol..

[106] Tanaka M., Szatmári I., Vécsei L. (2025). Quinoline quest: Kynurenic acid strategies for next-generation therapeutics via rational drug design. Pharmaceuticals.

[107] Ren T., Li D., Sun F., Pan L., Wang A., Li X., Bao Y., Zhang M., Zheng F., Yue H. (2025). Indole Propionic Acid Regulates Gut Immunity: Mechanisms of Metabolite-Driven Immunomodulation and Barrier Integrity. J. Microbiol. Biotechnol..

[108] Jazvinšćak Jembrek M., Oršolić N., Mandić L., Sadžak A., Šegota S. (2021). Anti-oxidative, anti-inflammatory and anti-apoptotic effects of flavonols: Targeting Nrf2, NF-κB and p53 pathways in neurodegeneration. Antioxidants.

[109] Petri S., Körner S., Kiaei M. (2012). Nrf2/ARE signaling pathway: Key mediator in oxidative stress and potential therapeutic target in ALS. Neurol. Res. Int..

[110] Rushworth S.A., MacEwan D.J. (2011). The role of nrf2 and cytoprotection in regulating chemotherapy resistance of human leukemia cells. Cancers.

[111] Kausar S., Wang F., Cui H. (2018). The role of mitochondria in reactive oxygen species generation and its implications for neurodegenerative diseases. Cells.

[112] Larosa V., Remacle C. (2018). Insights into the respiratory chain and oxidative stress. Biosci. Rep..

[113] Hardeland R. (2005). Atioxidative protection by melatonin: Multiplicity of mechanisms from radical detoxification to radical avoidance. Endocrine.

[114] Poeggeler B., Saarela S., Reiter R.J., Tan D.X., Chen L.D., Manchester L.C., Barlow-Walden L.R. (1994). Melatonin—A highly potent endogenous radical scavenger and electron donor: New aspects of the oxidation chemistry of this indole accessed in vitro a. Ann. N. Y. Acad. Sci..

[115] Poeggeler B., Sambamurti K., Siedlak S.L., Perry G., Smith M.A., Pappolla M.A. (2010). A novel endogenous indole protects rodent mitochondria and extends rotifer lifespan. PLoS ONE.

[116] Guo P., Pi H., Xu S., Zhang L., Li Y., Li M., Cao Z., Tian L., Xie J., Li R. (2014). Melatonin improves mitochondrial function by promoting MT1/SIRT1/PGC-1 alpha-dependent mitochondrial biogenesis in cadmium-induced hepatotoxicity in vitro. Toxicol. Sci..

[117] Beckhauser T.F., Francis-Oliveira J., De Pasquale R. (2016). Reactive oxygen species: Physiological and physiopathological effects on synaptic plasticity: Supplementary issue: Brain plasticity and repair. J. Exp. Neurosci..

[118] Ciszowski K., Gomółka E., Gawlikowski T., Szpak D., Potoczek A., Boba M. (2016). Brain-derived neurotrophic factor (BDNF) and nerve growth factor (NGF) blood levels in patients with acute carbon monoxide poisoning-a preliminary observations. Prz. Lek..

[119] Cunha C., Brambilla R., Thomas K.L. (2010). A simple role for BDNF in learning and memory?. Front. Mol. Neurosci..

[120] Thangwong P., Jearjaroen P., Govitrapong P., Tocharus C., Tocharus J. (2022). Melatonin improves cognitive function by suppressing endoplasmic reticulum stress and promoting synaptic plasticity during chronic cerebral hypoperfusion in rats. Biochem. Pharmacol..

[121] Singh A.A., Jo S.H., Kiddane A.T., Niyonizigiye I., Kim G.D. (2023). Indole-3-carbinol induces apoptosis in AGS cancer cells via mitochondrial pathway. Chem. Biol. Drug Des..

[122] Herring J.A., Elison W.S., Tessem J.S. (2019). Function of Nr4a orphan nuclear receptors in proliferation, apoptosis and fuel utilization across tissues. Cells.

[123] Chen B., Zhao J., Zhang R., Zhang L., Zhang Q., Yang H., An J. (2022). Neuroprotective effects of natural compounds on neurotoxin-induced oxidative stress and cell apoptosis. Nutr. Neurosci..

[124] Akhtar R.S., Ness J.M., Roth K.A. (2004). Bcl-2 family regulation of neuronal development and neurodegeneration. Biochim. Biophys. Acta (BBA)-Mol. Cell Res..

[125] Wang W.-Y., Tan M.-S., Yu J.-T., Tan L. (2015). Role of pro-inflammatory cytokines released from microglia in Alzheimer’s disease. Ann. Transl. Med..

[126] Kurhaluk N., Kołodziejska R., Kamiński P., Tkaczenko H. (2025). Integrative neuroimmune role of the parasympathetic nervous system, vagus nerve and gut microbiota in stress modulation: A narrative review. Int. J. Mol. Sci..

[127] Santucci N.E., Ferreira S.L. (2026). NR4A Receptors in Immunity: Bridging Neuro-Endocrine and Inflammatory Pathways. Receptors.

[128] An J., Chen B., Kang X., Zhang R., Guo Y., Zhao J., Yang H. (2020). Neuroprotective effects of natural compounds on LPS-induced inflammatory responses in microglia. Am. J. Transl. Res..

[129] Yang J., Song X., Yan S., Li Q., Yang W. (2025). The gut microbiota influences neurodegenerative diseases through the gut-brain axis: Molecular mechanisms and effects on immune function. Front. Immunol..

[130] Chiu Y.-J., Lin C.-H., Lin C.-Y., Yang P.-N., Lo Y.-S., Chen Y.-C., Chen C.-M., Wu Y.-R., Yao C.-F., Chang K.-H. (2023). Investigating therapeutic effects of indole derivatives targeting inflammation and oxidative stress in neurotoxin-induced cell and mouse models of Parkinson’s disease. Int. J. Mol. Sci..

[131] Corvino A., Caliendo G., Fiorino F., Frecentese F., Valsecchi V., Lombardi G., Anzilotti S., Andreozzi G., Scognamiglio A., Sparaco R. (2024). Newly Synthesized Indolylacetic Derivatives Reduce Tumor Necrosis Factor-Mediated Neuroinflammation and Prolong Survival in Amyotrophic Lateral Sclerosis Mice. ACS Pharmacol. Transl. Sci..

[132] Fatemeh G., Sajjad M., Niloufar R., Neda S., Leila S., Khadijeh M. (2022). Effect of melatonin supplementation on sleep quality: A systematic review and meta-analysis of randomized controlled trials. J. Neurol..

[133] Menczel Schrire Z., Phillips C.L., Duffy S.L., Marshall N.S., Mowszowski L., La Monica H.M., Stranks L., Gordon C.J., Chapman J.L., Saini B. (2024). 3-Month Melatonin Supplementation to Reduce Brain Oxidative Stress and Improve Sleep in Mild Cognitive Impairment: A Randomised Controlled Feasibility Trial. J. Pineal Res..

[134] Duan L., Li X., Ji R., Hao Z., Kong M., Wen X., Guan F., Ma S. (2023). Nanoparticle-based drug delivery systems: An inspiring therapeutic strategy for neurodegenerative diseases. Polymers.

